# Experimental data on the adsorption of Reactive Red 198 from aqueous solution using Fe_3_O_4_ nanoparticles: Optimization by response surface methodology with central composite design

**DOI:** 10.1016/j.dib.2018.07.008

**Published:** 2018-07-10

**Authors:** Mohammad Hadi Dehghani, Mina Pourshabanian, Zoha Heidarinejad

**Affiliations:** a Department of Environmental Health Engineering, School of Public Health, Tehran University of Medical Sciences, Tehran, Iran; b Institute for Environmental research, Center for Solid Waste Research, Tehran University of Medical Sciences, Tehran, Iran; c Department of Environmental Health Engineering, Faculty of Health, Hormozgan University of Medical Sciences, Bandar Abbas, Iran

**Keywords:** Reactive Red 198, Adsorption, Nanoparticles, Composite design

## Abstract

The aim of this study was to evaluate the efficiency of Fe_3_O_4_ nanoparticles for Reactive Red 198 adsorption. The adsorbents were characterized by SEM and XRD. In this dataset, the influence of Reactive Red 198 dye concentration, solution pH, adsorbent dosage, and contact time on Reactive Red 198 dye adsorption by Fe_3_O_4_ nanoparticles was tested by central composite design (CCD) under response surface methodology (RSM). The Fe_3_O_4_ nanoparticles adsorbent was prepared by chemical co-precipitation. The process efficiency was achieved in optimal conditions including pH=7, adsorbent dosage equal to 0.5 g/L, initial dye concentration of 100 mg/L, contact time equal to 30 min, 88%. Overall, the data offer a facile adsorbent to remove Reactive Red 198 dye from aqueous solutions.

**Specifications Table**

**Table:** 

Subject area	Environmental Chemistry
More specific subject area	Adsorption
Type of data	Table, image and figure
How data was acquired	– Design experiments were carried out using Central Composite Design (CCD) and adsorption tests were done in batch mode. The parameters were evaluated using RSM. – The concentrations of Reactive Red 198 in the samples were measured using a UV-visible spectrophotometer (HACH, USA, model DR6000) set at a wavelength 518 nm. – The characteristics of the nanoparticles were analyzed using SEM (Tuscan Mira 3 LMU) and XRD (Philips X’Pert, Netherlands).
Data format	Analyzed
Experimental factors	The data of effects of main experimental parameters including contact time, initial dye concentration, adsorbent dosage and solution pH were acquired.
Experimental features	The objective of this research were to i) prepare Preparation of Fe_3_O_4_ nanoparticles using chemical co-precipitation and ii) to study the Reactive Red 198 adsorption onto the Fe_3_O_4_ nanoparticles. iii) Optimization of Reactive Red 198 adsorption onto Fe_3_O_4_ nanoparticles adsorbent using RSM
Data source location	Tehran University of Medical Sciences, Tehran, Iran
Data accessibility	Data are available in the paper

**Value of the data**

• The dataset will be useful for the application of the produced Fe_3_O_4_ nanoparticles in the removal of Reactive Red 198 dye from water and wastewater.
• This data offers a simple and environmentally friendly method for preparation of adsorbent from Fe_3_O_4_ nanoparticles.
• This data article presents a user central composite design (CCD) combined with response surface methodology (RSM) to optimize Reactive Red 198 removal from aqueous solution using adsorption process.

## 1 Data

**Fig. 1:**
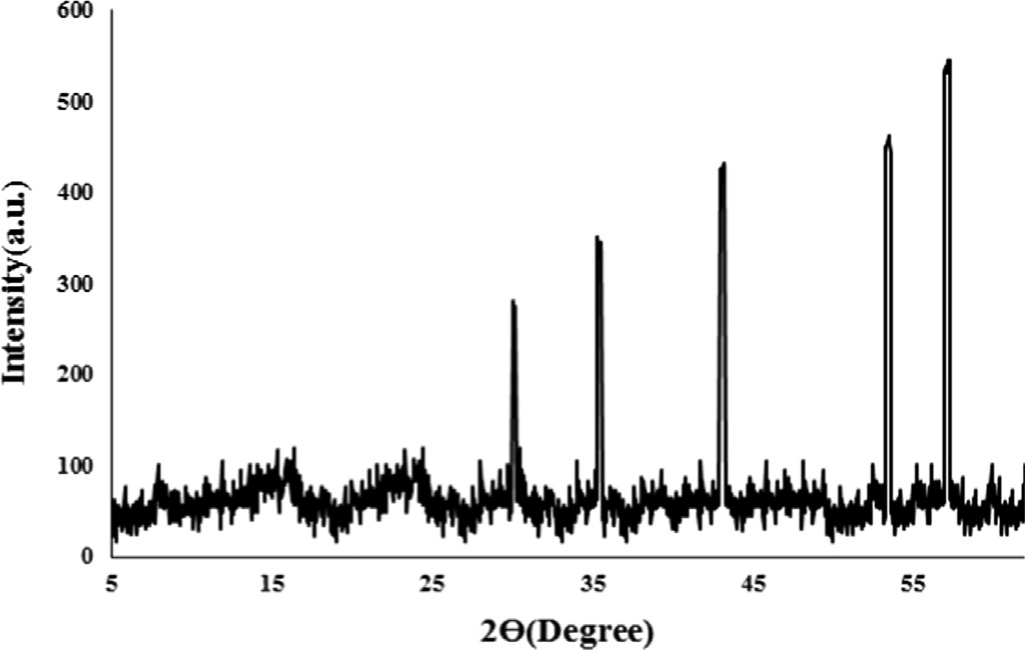
The XRD images Fe_3_O_4_ nanoparticles synthesized used adsorbent in the RR198 adsorption.

**Fig. 2:**
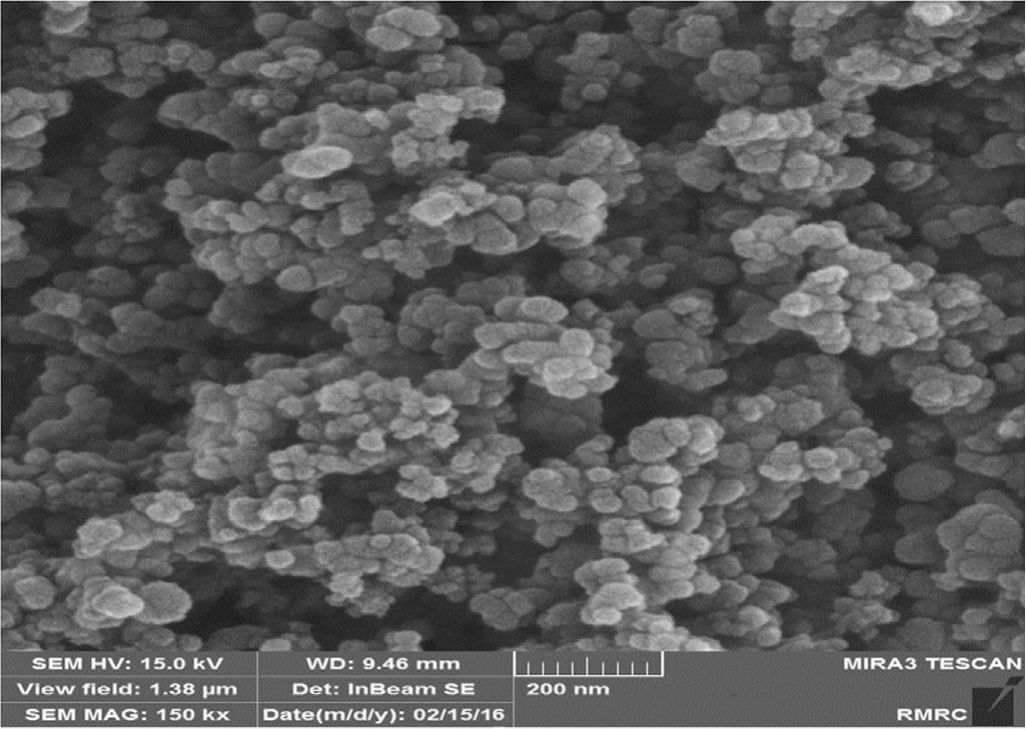
The SEM images Fe_3_O_4_ nanoparticles synthesized used adsorbent in the RR198 adsorption.

This dataset contains 3 Figures and 4 Tables. The XRD analysis of Fe_3_O_4_ nanoparticles is shown in Fig. 1. The SEM image of the prepared adsorbent is also illustrated in Fig. 2.

Structure and characteristics of a Reactive Red 198 (RR198) dye are seen in Table 1. The design matrix for the central composite designs (CCD) is listed in Table 2, Table 3.

**Table 1:** Structure and characteristics of RR198.

CAS number	18221
Type of dye	Azo
Chemical Formula	C_27_H_18_C_l_N_7_Na_4_O_15_S_5_
Molecular weight (g mol^-1^)	967.5
Maximum wavelength (λ_max_), nm	518
Molecular Structure

**Table 2:** Design matrix for the central composite designs.

**Factors**	**Low**	**Central**	**High**
pH	3	5	7
Time (min)	30	50	70
Concentration of dye (mg/L)	100	150	200
Adsorbent dosage (g/L)	0.25	0.37	0.5

**Table 3:** Design matrix for the CCD.

**Run**	**pH**	**Time (min)**	**Concentration of dye (mg/L)**	**Adsorbent dosage (g/L)**	**Efficiency (%)**
1	7	70	200	0.5	86
2	7	70	200	0.25	76
3	7	30	200	0.25	72
4	7	30	100	0.25	78
5	7	70	100	0.5	81
6	7	70	100	0.25	81
7	7	30	100	0.5	88
8	7	30	200	0.5	83
9	5	50	150	0.37	77
10	5	50	150	0.37	77
11	5	50	150	0.37	77
12	9	50	150	0.37	72
13	9	50	150	0.37	72
14	3	70	100	0.5	76
15	3	30	200	0.5	68
16	3	70	200	0.5	71
17	3	30	200	0.25	58
18	3	70	100	0.25	66
19	3	70	200	0.25	61
20	3	30	100	0.25	63
21	3	30	100	0.5	73
22	5	50	150	0.37	77
23	5	50	150	0.37	77
24	5	50	150	0.37	77
25	5	50	50	0.37	82
26	5	50	150	0.62	77
27	5	90	150	0.37	77
28	5	50	250	0.37	72
29	5	50	150	0.37	67
30	5	10	150	0.37	70
31	1	50	150	0.37	52
32	1	50	150	0.37	51
33	5	50	150	0.37	77
34	5	50	150	0.37	77
35	5	50	150	0.37	77

**Fig. 3:**
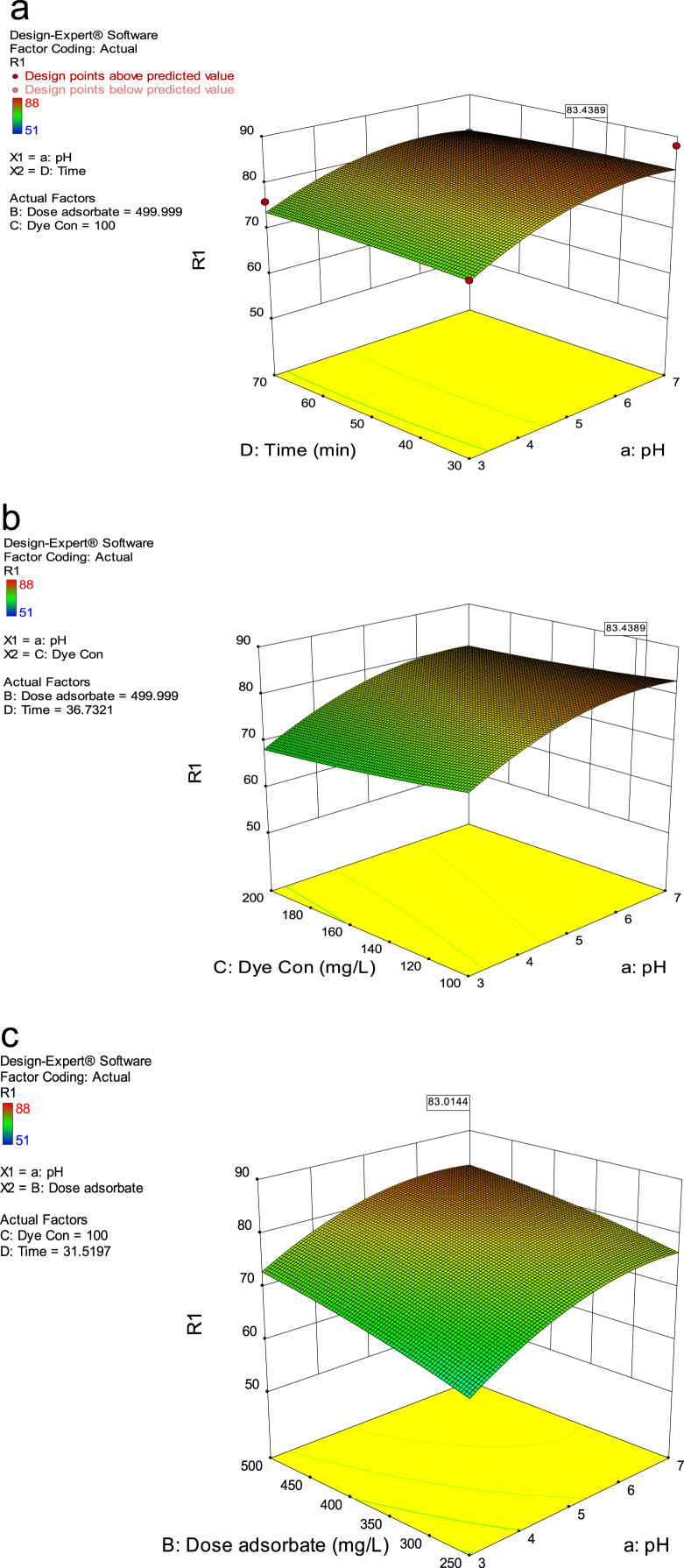
Central composite design 3-D surface plots showing effect of (a) pH and contact time, (b) pH and RR198 concentration, (c) pH and adsorbent dosage on Reactive Red 198 removal efficiency with the adsorbent.

The data for Analysis of variance (ANOVA) for second order model in the removal of RR198 show in Table 4. Central composite design 3-D surface plots which showing the effect of various parameters on RR198 removal efficiency with the adsorbent of Fe_3_O_4_ nanoparticles are presented in Fig. 3.

**Table 4:** Analysis of variance (ANOVA) for second order model in the removal of RR198.

Fixed Effects					
	Term	Error	*F*	*p*-value	
Source	df	df		Prob>*F*	
Whole-plot	2	6.07	71.77	<0.0001	Significant
a-pH	1	5.83	77.06	0.0001	
a^2	1	6.34	66.49	0.0001	
Subplot	12	14.42	12.73	<0.0001	Significant
B-Dose adsorbate	1	16.58	96.20	<0.0001	
C-Dye Con	1	16.58	30.22	<0.0001	
D-Time	1	16.58	9.77	0.0063	
aB	1	16.58	1.41	0.2515	
aC	1	16.58	1.41	0.2515	
aD	1	16.58	1.41	0.2515	
BC	1	16.58	2.11	0.1651	
BD	1	16.58	2.11	0.1651	
CD	1	16.58	2.11	0.1651	
B^2	1	8.54	2.45	0.1541	
C^2	1	8.54	0.93	0.3615	
D^2	1	8.54	0.65	0.4423

## 2 Experimental design, materials and methods

### 2.1 Materials

Reactive Red 198 dye powder with a purity of 99.6%, sulfuric acid, sodium hydroxide, FeCl_2_.6H_2_O, FeCl_3_.4H_2_O, ammonium hydroxide and ammonia were purchased from Merck and Sigma Aldridge companies.

### 2.2 Preparation Fe_3_O_4_ nanoparticles

Magnetic nanoparticles were prepared by chemical co-precipitation method. In this method, 16 mmol of FeCl_2_·6H_2_O and mmol 8 of FeCl_3_·4H_2_O with a stoichiometric ratio of 1 to 2 of Fe^2+^ and Fe^3+^ were mixed and in 200 ml of deionized water dissolved. The solution was then completely agitated at ambient temperature using a magnetic stirrer. Under these conditions, 10 ml of 25% ammonia was slowly added to the resulting solution, followed by a black colored sediment at the bottom of the container was formed. This precipitate indicates the presence of magnetic iron nanoparticles. After 10 min, stirring continuously to remove ammonia from the reaction medium, the iron nanoparticles were collected at the end of the container by placing the magnet under the reaction vessel, then the supernatant was removed and the precipitate was washed three times with ionizing water [1], [2], [3], [4], [5]. Finally, the characteristics of magnetic nanoparticles were determined by scanning electron microscopy (SEM) and X-ray diffraction (XRD).

### 2.3 Design of experiments

The pH of the solutions was adjusted prior to the adsorption by using 0.1 M solutions of HCl and NaOH and measured using a pH meter (Sense Ion 378, Hack) [6], [7], [8], [9], [10]. In this study, Design Expert 7.0.1 software was used to determine the number of experiments, values, and range of variables. The effects of operational parameters including pH (3–7), contact time (30–70 min), RR198 initial concentration (100–200 mg/L) and adsorbent dosage (0.25–0.5 g/L) on the amount of RR198 adsorbed onto the Fe_3_O_4_ nanoparticles were assessed and optimized by central composite design (CCD) combined with response surface methodology (RSM) was used to identify the optimum conditions for maximum removal of Reactive Red 198 dye [11], [12], [13]. The data were analyzed by the statistical method (ANOVA).

The removal efficiency, RE, (%) and equilibrium adsorption capacity, q_e_, (mg g^−1^) were calculated as follows [13]:

(1) $$\mathrm{RE}=\frac{(C_{i}-C_{t})}{C_{i}}\times 100$$

where *C_i_*, *C*_*e*,_ and *C_t_* are the initial, equilibrium and at time t of RR198 concentrations (mg/L), respectively.

After statistical analysis, the proposed model was presented as a second order equation in terms of actual parameters by software. This mathematical equation shows the adsorption rate of Reactive Red 198 dye by the adsorption process as a function of the amount of different operating parameters:

(2) $$\begin{array}{c} F\;\mathrm{removal}\;\left(\%\right):\;29.86979+\left(12.59375\times \text{pH}\right)+\left(0.074833\times \text{Adsorbent}\;\text{dose}\right)\\ –\left(0.20625\times \text{Dye}\;\text{Con}\right)+\left(0.22448\times \text{Time}\right) \end{array}$$
